# A Crowdsourced Physician Finder Prototype Platform for Men Who Have Sex with Men in China: Qualitative Study of Acceptability and Feasibility

**DOI:** 10.2196/13027

**Published:** 2019-10-08

**Authors:** Dan Wu, Wenting Huang, Peipei Zhao, Chunyan Li, Bolin Cao, Yifan Wang, Shelby Stoneking, Weiming Tang, Zhenzhou Luo, Chongyi Wei, Joseph Tucker

**Affiliations:** 1 Faculty of Infectious and Tropical Diseases London School of Hygiene and Tropical Medicine London United Kingdom; 2 Social Entrepreneurship to Spur Health Global Guangzhou China; 3 School of Public Health Emory University Atlanta, GA United States; 4 Shenzhen Nanshan Canter for Chronic Disease Control Shenzhen China; 5 Department of Health Behavior University of North Carolina at Chapel Hill Chapel Hill, NC United States; 6 School of Media and Communication Shenzhen University Shenzhen China; 7 School of Medicine University of North Carolina at Chapel Hill Chapel Hill, NC United States; 8 Project-China University North Carolina at Chapel Hill Guangzhou China; 9 Dermatology Hospital Southern Medical University Guangzhou China; 10 Department of Social and Behavior Health Sciences Rutgers School of Public Health Piscataway, NJ United States

**Keywords:** gay-friendly doctors, social media, crowdsourcing, prototype evaluation, men who have sex with men, China

## Abstract

**Background:**

Men who have sex with men (MSM), including both gay and bisexual men, have a high prevalence of HIV and sexually transmitted infections (STIs) in China. However, healthcare seeking behaviors and engagement in clinical services among MSM are often suboptimal. Global evidence shows that embedding online HIV or sexual health services into gay social networking applications holds promise for facilitating higher rates of healthcare utilization among MSM. We developed a prototype of a gay-friendly health services platform, designed for integration within a popular gay social networking app (Blued) in China.

**Objective:**

The purpose of this study was to evaluate the acceptability of the platform and ask for user feedback through focus group interviews with young MSM in Guangzhou and Shenzhen, cities in Southern China.

**Methods:**

The prototype was developed through an open, national crowdsourcing contest. Open crowdsourcing contests solicit community input on a topic in order to identify potential improvements and implement creative solutions. The prototype included a local, gay-friendly, STI physician finder tool and online psychological consulting services. Semistructured focus group discussions were conducted with MSM to ask for their feedback on the platform, and a short survey was administered following discussions. Thematic analysis was used to analyze the data in NVivo, and we developed a codebook based on the first interview. Double coding was conducted, and discrepancies were discussed with a third individual until consensus was reached. We then carried out descriptive analysis of the survey data.

**Results:**

A total of 34 participants attended four focus group discussions. The mean age was 27.3 years old (SD 4.6). A total of 32 (94%) participants obtained at least university education, and 29 (85%) men had seen a doctor at least once before. Our survey results showed that 24 (71%) participants had interest in using the online health services platform and 25 (74%) thought that the system was easy to use. Qualitative data also revealed that there was a high demand for gay-friendly healthcare services which could help with care seeking. Men felt that the platform could bridge gaps in the existing HIV or STI service delivery system, specifically by identifying local gay-friendly physicians and counselors, providing access to online physician consultation and psychological counseling services, creating space for peer support, and distributing pre-exposure prophylaxis and sexual health education.

**Conclusions:**

Crowdsourcing can help develop a community-centered online platform linking MSM to local gay-friendly HIV or STI services. Further research on developing social media–based platforms for MSM and evaluating the effectiveness of such platforms may be useful for improving sexual health outcomes.

## Introduction

HIV prevalence has steadily increased among men who have sex with men (MSM) in China. According to a national estimate, the prevalence increased from 1.2% in 2003 to 7.8% in 2016 [1]. However, healthcare seeking behaviors and engagement in clinical services among MSM are often suboptimal. Many MSM living with HIV in China have poor retention along the HIV care continuum [2]. Limited knowledge of HIV and HIV care, stigma against people living with HIV and against nonheterosexual practices, and lack of navigation guidance in the health system are commonly identified factors that contribute to attrition along the HIV care cascade [3-6]. A Chinese study also revealed men’s concerns about inadvertent disclosure of their sexual orientation [7].

Electronic health technologies (ie, electronic Health [eHealth]) have become a common platform for behavioral interventions because of their wide coverage, convenience, and low cost [8]. eHealth programs use an electronic device or information technologies to provide their services [9]. Among MSM, eHealth interventions may be even more appealing because of the anonymity and the protection of their confidentiality.

Many MSM in China use the internet to find sexual partners, creating an opportunity for online sexual health promotion [10]. Several studies suggest that MSM are interested in using eHealth tools for HIV prevention or sexual health promotion and would be willing to share personal information on the use of such apps [11,12]. A cross-sectional survey of MSM in the United States in 2015 found that over 80% of the participants had an interest in embedding tools for finding lesbian, gay, bisexual, transgender, and queer (LGBTQ)-friendly doctors, and for directly consulting with physicians, into their social apps [13]. Lists of LGBTQ-friendly physicians are now available in the United States [14] and several other high-income countries [15,16]. However, there has not been as much research done on the development of eHealth interventions that help men find gay-friendly physicians in middle-income countries.

Given the need for improving MSM’s linkage to services, we developed a prototype of a platform designed to be embedded within a popular gay social app (Blued) in China. The goal of the platform was to facilitate higher rates of healthcare utilization among MSM by identifying local gay-friendly health services. The platform presents Blued users with a list of gay-friendly doctors endorsed by community members who have used their services. The platform allows users to seek online counseling or schedule in-person appointments. The purpose of this study was to evaluate the acceptability of the platform and ask for user feedback through focus group discussions with young MSM in two major cities in Southern China.

## Methods

### Participant Recruitment

Both convenience and purposive sampling strategies were used to recruit men who were willing to share their experiences and perspectives. By using a qualitative approach, we did not aim to generalize the study results to the wider community but instead to have a better understanding of some key issues. In focus group discussions, group interactions and dynamics facilitate rich information generation, which can help establish a range of perceptions on a topic of interest [17]. Thus, we conducted a total of four focus groups, each made up of 8-10 MSM, to evaluate the platform. Two focus groups were conducted in Shenzhen and two were conducted in Guangzhou, both cities in Southern China's Guangdong Province. Men were recruited through local community-based organizations (CBOs) that work with MSM in Shenzhen and Guangzhou. Recruitment was conducted both on-site and via an announcement on WeChat, an instant messaging mobile app which has a widespread posting function like Twitter. Participants were considered eligible for the study if they met the following criteria: (1) aged 18 years or older; (2) born biologically male; (3) have had sex with a man at least once; and (4) currently residing in Guangzhou or Shenzhen, China. Other purposive sampling criteria included whether they disclosed their sexual orientation to others (yes/no), whether they had ever been tested for HIV before (yes/no), and past experiences of seeing a physician (within 1 year, 1-2 years, 2-5 years, and never saw a physician before).

### Platform Development

**Figure 1:**
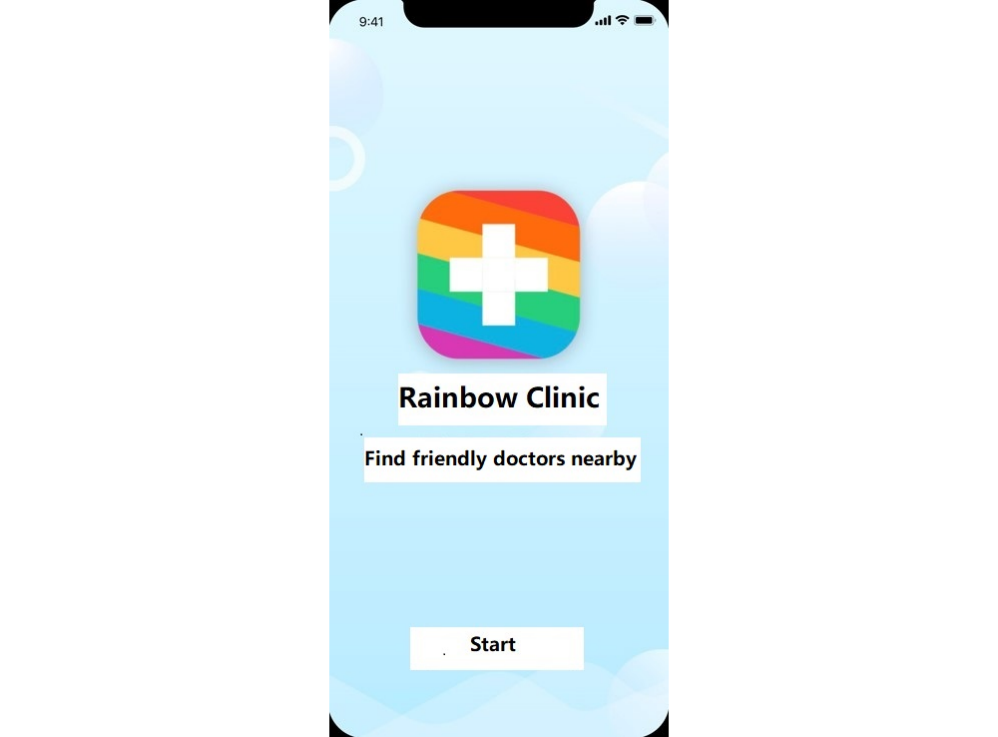
Start page of the prototype.

The prototype of the health-seeking mobile platform was developed based on submissions to an open crowdsourcing contest, which was held from March 1, 2018 to March 25, 2018. Open crowdsourcing contests ask for community input on a topic to identify potential improvements and implement creative solutions [18,19]. The contest was promoted through social media announcements on WeChat. We solicited submissions about the function, content, and design of the platform, but specifically, we collected ideas for the name, logo, slogan, and features of the platform. Figure 1 shows the start page of the prototype, which includes the name, logo, and slogan. Submissions were voted on by the public through social media and then evaluated by a panel consisting of public health researchers, a representative from a local LGBT CBO, and a Blued developer. Using the winning name, slogan, logo, and function content, we created a prototype for a health seeking platform tailored for MSM. Please see Multimedia Appendix 1 for details on the contest.

The mobile platform, as the most exceptional design product of the contest, had the following main functions: (1) it could identify local sexually transmitted infections (STI) physician services; (2) it provided online psychological consulting services; and (3) it created a network for peer communication and support. The local physician finder connected users to nearby gay-friendly physicians, and users could sort and view physician profiles by specialty (HIV and other STIs), user recommendations, and distance from their location. For the consulting feature, users could choose different forms of service delivery, including online, via phone call, and via an in-person appointment. The psychological consulting service feature aimed to provide support and mental health care for MSM navigating life as a sexual minority in China (eg, psychologists might provide advice on methods for disclosing sexual orientation to friends and family). Peer communication and support aimed to facilitate peer-led online communications within the platform, and users could anonymously ask and answer questions about sexual health, coming out, health services, and so on.

### Focus Group Discussion

The focus group discussions were conducted in a private conference room and facilitated by two researchers experienced in qualitative research. Each interview lasted between one and two hours. Snacks and drinks were provided to ease participant burden and build rapport. We explained the purpose of the study and stressed confidentiality to participants, and written consent was obtained before the focus group discussion began. Then, we played a video showing the features of the online healthcare seeking platform and asked the men to share their reactions (see Multimedia Appendix 2). The focus group discussions focused on evaluating the acceptability and feasibility of the early product of the crowdsourcing contest rather than the contest method. A topic guide was used to guide the overall structure of the interview, but discussions were kept relatively open, and participants were invited to provide any feedback they thought might be relevant for improving the platform. A short survey on the acceptability and usability (perceived ease of use) of the platform was collected following the focus group discussions. Perceived ease-of-use is an important construct of usability and positively affects user acceptability of information technology [20]. The survey was used to triangulate the qualitive findings as an approach to enhance validity. Each participant received a small monetary award of ¥50 (7.50 United States Dollars) for their participation. We ceased focus group discussions once we reached thematic saturation.

### Data Analysis

The focus group discussions were audio-recorded and transcribed, then a thematic analysis of the transcripts was conducted [21]. We developed an initial code framework based on the interview guide and applied this code to the first interview. In addition to predetermined codes, during the first coding process we also used an inductive coding approach to allow unexpected codes to emerge from the raw data. The revised codebook, consisting of predefined and inductive-derived codes, was then applied to the remaining transcripts. All transcripts were double coded by DW and PZ, and discrepancies were discussed with a third independent individual (WH). We then read and organized the coded text to find themes and subthemes, and selected relevant quotes were then translated into English. Nvivo 12 software (QSR International, Melbourne) was used to carry out all qualitative data analysis. Descriptive analysis was conducted to analyze survey data using SPSS version 25 (IBM, New York).

### Ethical Statement

This study received approval from institutional review boards at the University of North Carolina at Chapel Hill and Southern Medical University Dermatology Hospital.

## Results

### Participant Characteristics

A total of 34 participants attended four focus group discussions. Table 1 shows the participants characteristics. The mean age was 27.3 years old (SD 4.6). At the time of the focus group discussions 18 (53%) men were living in Guangzhou and 16 (47%) men were living in Shenzhen. A total of 33 (97%) individuals self-identified as male and one self-identified as female. In addition, 32 (94%) had obtained at least a university education, 7 (21%) men were students, and 20 (59%) earned an annual salary of $14,052 or less. Overall, 27 (79%) men took at least one HIV test in the past 12 months, and 29 (85%) men had seen a doctor at least once before. Among them, 18/29 (62%) had never disclosed their sexual orientation to health professionals and 15/29 (52%) were satisfied with their last physician encounter.

**Table 1:** Demographic characteristics of focus group discussion participants in China, 2018 (N=34).

Characteristics		Value
Age in years (mean, SD)		27.3 (4.6)
**City, n (%)**		
	Guangzhou	18 (53)
	Shenzhen	16 (47)
**Gender identity, n (%)**		
	Male	33 (97)
	Female	1 (3)
**Sexual orientation, n (%)**		
	Men who only have sex with men	32 (94)
	Bisexual	1 (3)
	Not sure	1 (3)
**Highest education, n (%)**		
	High school	2 (6)
	University	27 (79)
	Postgraduate	5 (15)
**Annual income (USD), n (%)**		
	<3516	6 (18)
	3516-8784	7 (21)
	8784-14052	7 (21)
	>14052	14 (41)
**Employment status, n (%)**		
	Student	7 (21)
	Employed	26 (77)
	Unemployed	1 (3)
**Ethnicity, n (%)**		
	Han	32 (94)
	Non-Han	2 (6)
**Number of HIV tests done in past 12 months, n (%)**		
	0	7 (21)
	1	10 (29)
	2	11 (32)
	4 or above	6 (18)
**Last time saw a physician, n (%)**		
	Within 1 year	25 (74)
	1-2 years	3 (9)
	2-5 years	1 (3)
	Never	5 (15)
**Sexual orientation disclosure to your physician (N=29), n (%)**		
	Yes	11 (38)
	No	18 (62)
**Degree of satisfaction with last experience of seeing a doctor time (N=29), n (%)**		
	Very satisfied	5 (17)
	Satisfied	10 (35)
	Neutral	14 (48)

### Acceptability

Table 2 shows participants’ responses to the survey questions. We found that 24/34 (71%) participants showed interest in using an online health services platform, and the prototype of the platform we presented was highly acceptable to our participants. Qualitative interviews revealed that there was a high demand for gay-friendly healthcare services, and men commented that a platform linking online and offline gay-friendly healthcare services would encourage healthcare seeking behaviors.

Further, privacy and confidentiality were considered to be well protected by the security measures of the platform. For example, participant 2 from group one in Shenzhen commented that the:

two most important features of this platform are privacy protection and gay-friendly services.

Allowing a user to seek health information securely and anonymously with this online platform was particularly helpful to alleviate fears of being discriminated or judged (see Multimedia Appendix 3, Box 1, Quote 1).

Participants expected that such a platform would help expand men’s access to in-person health services. One man said that:

This platform is meaningful because if I know that there are gay-friendly services in other hospitals, I don’t have to confine my access in one specific hospital.

Another participant from the same group mentioned that such a platform was a necessity for those who had already developed relevant STI symptoms and felt anxious about finding out the cause (see Multimedia Appendix 3, Box 1, Quote 2).

**Table 2:** Acceptability and perceived usability of the online health care seeking prototype platform by MSM^a^ in China, 2018 (N=34). All values are given as n (%).

Acceptability and perceived usability items	Strongly agree	Agree	Neutral	Disagree	Strongly disagree
I am willing to use this platform frequently	7 (21)	17 (50)	7 (21)	3 (9)	0 (0)
I think this platform is unnecessarily complicated	3 (9)	5 (15)	10 (29)	15 (44)	1(3)
I think this platform is easy to use	6 (18)	19 (56)	8 (24)	0 (0)	0 (0)
I think that I would need the support of a technical person to be able to use this system	1 (3)	0 (0)	2 (6)	16 (47)	15 (44)
I find the various functions in this system are well integrated	6 (18)	11 (32)	16 (47)	1 (3)	0 (0)
I think there is too much inconsistency in the system	3 (9)	2 (6)	11 (32)	17 (50)	1 (3)
I would imagine that most people would learn to use this system very quickly	10 (29)	18 (53)	4 (12)	2 (6)	0 (0)
I find the system very cumbersome to use	0 (0)	0 (0)	2 (6)	22 (65)	10 (29)
I feel very confident using the system	12 (35)	16 (47)	4 (12)	1 (3)	0 (0.0)
I think I need to learn a lot of things before I can get going with this system	1 (3)	2 (6)	4 (12)	17 (50)	9 (27)

^a^MSM: men who have sex with men.

### Perceived Usability

About three quarters (25/34, 74%) of men thought that the system was easy to use and only one (3%) commented that he would need technical support to be able to use it. Half of them regarded the various functions of the platform as well integrated. Most men (28/34, 82%) felt confident about using it.

### Specific Functions of the Platform

We asked for participants’ views towards the functions of the prototype, specifically about physician consultation services, psychological support and consulting services, and peer communication and support. Participants tended to view peer communication and support as a part of psychological counselling services. Additionally, we found that men were interested in pre-exposure prophylaxis (PrEP) services and sexual health education.

### Physician Consultation Services

Participants discussed the various forms and timing of service delivery, quality of services, and desirable types of health services. Many men showed preferences for audio or video consultations compared to text-image consultations, as the former was thought to be more efficient (Multimedia Appendix 3, Box 2, Quotes 1 and 2). Others felt a combination of all forms would be most acceptable (Multimedia Appendix 3, Box 2, Quote 3). Participants expressed concerns about delayed service delivery via the platform due to the demands of a physicians’ schedule (Multimedia Appendix 3, Box 2, Quote 4). Appointment services may mitigate such concerns and would be important for ensuring prompt service delivery (Multimedia Appendix 3, Box 2, Quote 5). In terms of quality of services, the qualifications of physicians and recognition by a public medical authority were the most essential considerations for participants (Multimedia Appendix 3, Box 2, Quotes 6 and 7).

Men expressed a desire for services relevant to sexual health and homosexual practices from the platform. Several particular services were preferred, including HIV or STI testing and treatment, anorectal services, urology, and relevant dermatological services (Multimedia Appendix 3, Box 2, Quote 8). One group in Shenzhen described that long-term, follow-up services were especially necessary for elderly patients and those living with chronic conditions, such as people living with HIV (Multimedia Appendix 3, Box 2, Quote 9 and 10).

### Psychological Support and Consultations

To facilitate a more comprehensive analysis, we divided this issue of psychological services into peer support and professional consultations. Participants acknowledged that there is a great need for mental health care, but that gay-friendly psychological services were lacking (Multimedia Appendix 3, Box 3, Quotes 1 and 2). Some attributed nondisclosure of sexual orientation to the health professions’ lack of capacity to deal with mental health issues (Multimedia Appendix 3, Box 3, Quote 3). A man from group 1 in Guangzhou said that:

You can provide peer counselling first, then move to professionals.

Participant 2

According to the same participant, peer counselling was “free and easier to find”, while “professional consultation services are more expensive”. Another participant from group 1 responded:

They (professionals) are standing in a high position and looking down, and we would feel pressure or even be subject to discrimination, which makes it worse.

Participant 1

However, other men from group 2 in Guangzhou disagreed, saying that “peers are far from professional” (Participant 1). Professional psychological consultations were considered to be most useful as a resource for the coming out process and for treating mental disorders such as depression (Multimedia Appendix 3, Box 3, Quotes 4 and 5).

### Pre-Exposure Prophylaxis

Additionally, although not an aspect of the prototype design, men mentioned the necessity of providing both educational information and accessibility for PrEP. Group 1 in Guangzhou mentioned that “both PrEP and PEP services should be strengthened” and this platform could be a good channel for promotion and education. However, risk compensation, like having more condomless sex, was also recognized by our participants (group 1 in Guangzhou).

### Sexual Health Education

Health education about HIV and sexual health was thought to be an important platform function. This function is particularly important for those who have recently identified as gay and have limited knowledge about safe sex practices. Men suggested that such a feature include information about HIV, methods of HIV prevention, and other safe sex practices (Multimedia Appendix 3, Box 4, Quotes 1 and 2). Providing educational information about HIV might generate interest in using the platform because of its relevance to potential users’ daily lives (Multimedia Appendix 3, Box 4, Quote 3). They also showed interest in information about coping with HIV infection and navigating life either as, or with, a partner living with HIV (Multimedia Appendix 3, Box 4, Quote 4).

## Discussion

### Principal Results

Many MSM delay seeking necessary healthcare and have difficulty finding gay-friendly physician services, and few interventions focus on linking MSM to local health services in low-and-middle income countries. We collected qualitative and survey data to evaluate men’s acceptability and usability of a crowdsourced social media–based eHealth intervention tailored to MSM. This study extends the literature by evaluating men’s acceptability of an online health service prototype that aims to link MSM to local health services in China.

We found that an online healthcare platform which links online to offline services was highly acceptable among our participants. This is consistent with previous research in China [12] and the United States [22]. We identified three main reasons for strong interest in the platform. First, as an online platform tailored to MSM, it provided a channel for anonymously seeking sexual health services, which may have mitigated some of the hesitation about using facility-based services [23], as men are fearful of health professional discrimination in traditional clinics [24]. Second, a platform where men can comfortably disclose their sexual history holds promise for reducing such fears and facilitating help seeking. Third, the crowdsourced platform provided a mechanism to compile all local, gay-friendly, sexual, and psychological health services into a single platform. This simplified the process of finding a gay-friendly doctor.

We also found that most men felt the platform was easy to use and were confident in their ability to use it, which is like findings from previous literature on eHealth platforms supporting HIV patient self-management [25]. Levels of ease-of-use in health information technology significantly affect a customer’s intention to engage [26], and it is important for MSM to continue using a health app over time [27]. We developed the prototype platform based on our previous crowdsourcing contests. Crowdsourcing is an effective approach to improve community engagement in sexual health promotion and HIV prevention programs [28,29]. This community-centered platform design approach may contribute to a user-friendly health information product; however, another reason may be that our participants were young adults who were well educated and skilled at using complicated products. The usability of the prototype platform among elderly groups, those with poor health literacy, or those with little experience with using mobile phones is still unknown.

Men in our study desired help in finding physicians and services related to PrEP. This is consistent with earlier findings of high demand for PrEP among MSM in China [30]. PrEP services are not currently approved by the China Food and Drug Administration and are largely unavailable in the country, but several pilot programs in developed cities suggest that there is growing demand [31]. Strong evidence has confirmed the efficacy of PrEP in preventing HIV acquisition in key populations [32], and the World Health Organization recommends PrEP for use in MSM [33]. Thus, incorporating information about PrEP into eHealth platforms may be useful.

### Limitations

Our study has several limitations. First, we evaluated men’s acceptability and perceived usability by describing the platform, showing a short video, and providing a PowerPoint presentation. Men were not able to download the platform within the Blued environment, however, acceptance of various platforms was found, in earlier research, to be correlated to actual eHealth use [34]. Second, a qualitative study design has limited generalizability. Quantitative evaluations are needed to further determine community acceptance. Additionally, our focus group discussions only recruited men from two Chinese cities. Our subjects were mainly young, had higher education, and had a higher income. Thus, the results may not reflect views of MSM with different sociodemographic backgrounds and in other social contexts. Finally, we assessed one major construct of usability and did not evaluate other aspects such as learnability, operability, and understandability. Usability should be more comprehensively evaluated when the platform becomes available for men to use.

### Implications

More research is called for to help develop eHealth programs to meet MSM’s needs for sexual health services. Linking local, gay-friendly health services to MSM via social media platforms may be a promising approach to encourage necessary healthcare seeking. Further developing the platform, monitoring actual use by MSM, and refining the platform are also worthy of exploration. In addition, PrEP services are an important strategy to prevent HIV spread and the men in our study showed a great interest in PrEP, which both indicates a need to introduce it into China’s healthcare system and the establishment of supportive policies. Preparing men for PrEP by incorporating relevant information and services into the platform is also worthy of consideration. Meanwhile, integrating a gay-friendly physician finder with an MSM social networking app may introduce ethical concerns, as approval from the Chinese Food and Drug Administration is necessary for online health service provision. In summary, developing platforms that can be embedded into social media apps for MSM may be useful for improving the HIV care cascade and sexual health.

## Appendix

Multimedia Appendix 1 Crowdsourcing Contest for the Prototype Development.

Multimedia Appendix 2 Focus group discussion guide.

Multimedia Appendix 3 Focus group discussion quotations.

## Abbreviations

CBO: community-based organization
eHealth: electronic health
LGBTQ: lesbian, gay, bisexual, transgender, and queer
MSM: men who have sex with men
PrEP: pre-exposure prophylaxis
STIs: sexually transmitted infections
