# Supplementary material for: A Crowdsourced Physician Finder Prototype Platform for Men Who Have Sex with Men in China: Qualitative Study of Acceptability and Feasibility
Source: JMIR Public Health Surveill. 2019 Oct 8;5(4):e13027. doi: 10.2196/13027 (PMC6913768; doi:10.2196/13027)
Supplement: Multimedia Appendix 1 [file publichealth_v5i4e13027_app1.pdf]

Crowdsourcing Contest for the Prototype Development

Contest Procedure

The prototype of the health-seeking platform tailored for MSM was developed based on a crowdsourcing contest which was held from March 1st to March 25th, 2018 in China. We solicited content of the health-seeking platform prototype on the following four parts: name, slogan, logo, and functions. Participants can submit separate entries about all four or any part(s) of it. Consistency was evaluated if they submitted more than one part.

The advertisements of the contest were posted online through WeChat (an instant messaging and social mobile app similar to Facebook) official accounts as well as on Blued, the most popular gay dating app in China. An in-person event in Shenzhen University was organized to promote the contest offline. The in-person event included an introduction of the contest, guidance of submission, and a lecture of sexual health.

Crowdsourced entries were evaluated by crowd and expert judges. The crowd voted for their favorite entries online via the WeChat official account where all the entries were posted. The expert panel consisted of five professionals from the Shenzhen Center for Disease Control (CDC), community-based organizations, technology companies and universities. Experts evaluated and scored the submission based on pre-defined criteria, including relevance, understandability, aesthetic, novelty, consistency, elaboration, and popularity. Popularity was determined by the number of crowd votes. The evaluation criteria were shown in table 1. The results of the crowdsourcing contest were announced via the WeChat official account. Finalists were celebrated, and prizes were rewarded. We commended all participants for their contributions to the contest.

Table 1: The evaluation criteria and range of scores

Content	Evaluation Criteria	Range of Scores	Range of Total Score
Name	Relevance	1-4	4-15
	Understandability	1-4	
	Consistency	1-4	
	Popularity	1-3	
slogan	Relevance	1-4	4-15
	Understandability	1-4	
	Consistency	1-4	
	Popularity	1-3	
Logo	Aesthetic	1-10	5-50
	Relevance	1-10	
	Consistency	1-10	
	Understandability	1-10	
	Popularity	1-10	

Functions	Relevance	1-4	5-20
	Elaboration	1-4	
	Novelty	1-4	
	Clarity	1-4	
	Popularity	1-4	

Results

The online advertisements had a total of 8725 views during the contest. Overall, there were 103 entries from 73 contestants. Some contestants submitted two or more entries. Most of the contestants (n=69, 89.6%) were from Shenzhen city. More than half (n=44, 57.1%) of the contestants were male, and 18 of them self-identified as gay men.

Of the 103 entries, 71 contained names and slogans, 56 contained logos, 55 contained features of functions. The voting website had 14295 visits and 4294 votes in total. Ten entries had over 100 votes. The first place of the name, slogan and logo were used in the design of the platform prototype. All the submissions on the features of functions were collected and taken into consideration when we developed the platform prototype.
