# Supplementary material for: A Crowdsourced Physician Finder Prototype Platform for Men Who Have Sex with Men in China: Qualitative Study of Acceptability and Feasibility
Source: JMIR Public Health Surveill. 2019 Oct 8;5(4):e13027. doi: 10.2196/13027 (PMC6913768; doi:10.2196/13027)
Supplement: Multimedia Appendix 2 [file publichealth_v5i4e13027_app2.pdf]

Focus Group Discussion Guide[1]

Introduction: This study will collect feedback from men who have sex with men (MSM) on the Gay-Friendly Physician Finder Prototype Platform's content, format, and use. It would be used to assess men's acceptability and feasibility of the platform.

Participants: In order to participate in this focus group discussion, individuals must meet the following criteria:

- Age 18 years (age of majority in China) or older
- Born biologically male
- Have had sex with a man at least once
- Currently living in Shenzhen or Guangzhou, China

*We will attempt to include men who have disclosed their sexual orientation (or not), men who have HIV tested (and those who have not).

[we will be showing men the function of the prototype platform for the first time at the FGD so that they won't have exposure to this platform before the discussion]

Consent: Participants will be required to sign a consent form prior to participating in the focus group discussion. Each participant will receive a copy of the consent form for their records, and another copy will be kept by the facilitator. The facilitator will ask for consent from all participants before any audio-recording for data collection.

Demographics: Anonymous demographic data will be collected from focus group participants in the form of a simple questionnaire. The questionnaire will be provided to each participant at the end of the focus group discussion. This data will be kept in a secure location.

Facilitator: Knowledge of the skills that make an effective facilitator is essential to the success of a focus group discussion. Dr. Richard Krueger of the University of Minnesota has produced some training materials on focus group interviews, which may be helpful in the preparation of focus groups. Please see: http://www.eiu.edu/ihec/Krueger-FocusGroupInterviews.pdf

Discussion guide: This discussion guide provides a framework for the focus group discussion by highlighting the topics that need to be covered. The intent, however, is not for it to be used rigidly, like a questionnaire. During the focus group discussion, the facilitator should encourage in-depth deliberation of the subject matter, and encourage participants to take time to reflect, as well as to raise their own topics or questions for discussion.

Data collection: If all participants consent, the discussions will be recorded, transcribed for analysis. Additionally, participants will be asked to complete a short anonymous survey. The recordings will be securely stored until transcribed and then destroyed. The transcription will not contain information that would allow individuals to be linked to statements made during the discussion. Confidentiality will be strictly protected except in cases where disclosure is mandated by a court of law.

Logistics: The focus group will last about two hours with planned breaks. Participants will receive clear details about location, duration, date, and time well in advance of the discussion.

---

[1] This guide was created using the framework provided by a WHO template. The template can be found at http://www.who.int/patientsafety/implementation/checklists/instructions_focus_group.doc

Demographic information survey

Please fill in the blanks or circle the response for multiple choice questions.

1. Age: ___________

2. Your sexual identity: ☐ male ☐ female ☐ transgender ☐ others

3. Your sexual orientation: ☐ homosexual ☐ heterosexual ☐ bisexual ☐ others

4. What's your highest level of education that you have completed?

☐ primary school

☐ high school

☐ University

☐ Postgraduate

5. What's your monthly income?

☐ < 1500 RMB

☐ 1501 – 3000 RMB

☐ 3001 – 5000 RMB

☐ 5001 – 8000 RMB

☐ >8000 RMB

6. What's your occupation: _____________

7. What's your ethnicity: _____________

8. Number of HIV tests did you have in the past 12 months _____________

9. When is your last time visiting a physician?

☐ Within one year

☐ 1-2 years

☐ 2-5 years

☐ Never

10. Did you disclose your sexual orientation when you visited the physician last time?

☐ Yes

☐ No

☐ Can’t remember

11. How satisfied were you with the experience of seeing a physician last time?

☐ Very satisfied

☐ Satisfied

☐ Neutral

☐ Unsatisfied

☐ Very unsatisfied

Platform usability survey

	Strongly agree	Agree	Neutral	Disagree	Strongly disagree
1. I am willing to use this platform frequently.	1	2	3	4	5
2. I think this platform is unnecessarily complex.					
3. I think this platform is easy to use.					
4. I think that I would need the support of a technical person to be able to use this system					
5. I find the various functions in this system are well integrated.					
6. I think there is too much inconsistency in the system					
7. I would imagine that most people would learn to use this system very quickly					
8. I find the system very cumbersome to use					
9. I feel very confident using the system					
10. I think I need to learn a lot of things before I can get going with this system					

Discussion Guide

Welcome, introduction and instructions

Welcome: Welcome and thank you for agreeing to take part in this focus group. You have been asked to participate as your point of view is essential to the success of this project. We realize you lead busy lives and appreciate your time.

Introduction: [Facilitator will introduce his/her role in the project]. This focus group discussion is designed to understand your thoughts and feelings about the beta version of a mobile prototype platform which aims to connect users with gay-friendly services in their communities. The goal of our research is to improve the mobile prototype platform with your feedback. We hope that, in identifying gay-friendly physicians, the app will help users easily find friendly health services in local healthcare settings and improve healthcare utilization among the community in China. The focus group discussion will take approximately two hours.

Confidentiality: We want to assure you that everything you said during this discussion will be kept anonymous. Having said that, I want to ask everyone here if I may record the discussion in order to facilitate accurate data collection. The recording will remain secure in a locked facility until it is transcribed, at which point the recording will be destroyed. The transcription will not contain any identifiable information that will allow a person to be linked to their statements. [If yes, turn on the recorder]. We also ask that everyone in this room refrain from discussing other participant's comments outside of this focus group discussion. We want to encourage each participant to be as involved and honest as possible; however, if there are questions or discussions which you do not want to contribute to, you do not have to.

Ground rules

- Be respectful of your fellow participants, only one person should speak at a time.
- There is no set order in which people should speak, so please speak up if you have something you wish to share as it is important to collect opinions from all participants.
- The facilitator will try and make sure that everyone who wants to can speak their minds on each topic; however, in the interest of time and covering the essential topics the facilitator may ask that the group to move onto the next discussion point before all comments have been made.
- There is not a rigid format to this focus group; participants may pose their own discussion topics to the group at any time.
- There are no right or wrong answers. Dissenting viewpoints are expected and encouraged, but please refrain from attacking other's opinions or making personal attacks. For example, if someone disagrees with an idea they might simply state "I disagree because…", one shouldn't insult other person's opinions by saying something like "That idea is stupid because….".

**Are there any questions before we begin?*

Warm up

- To start, I'd like invite everyone to think of a favorite person and use that name for yourself during the focus group discussion. Let's go around the table.
- Take a couple of minutes to think about the last time you went to see a doctor and disclosed your sexual orientation. Would anyone be willing to talk about that experience?
    - Did this experience leave you with positive, negative, or neutral feelings?

- Has anyone ever tried to identify an MSM-friendly physician? How did you go about doing this?
- Now think about the last time you saw a doctor for sexual health related issue, but did not tell the doctor that you are MSM. Would anyone be willing to talk about that experience?
    - Did this experience leave you with positive, negative, or neutral feelings?

[guided tour of the function using a projector that shows mockups. This will include the features, name, logo, language, functions, design of the prototype. We will explain that the function will be embedded within Blued]

Guiding questions

- What are your initial impressions of the functions?
- Which function(s) did you find to be the most useful?
- Were there functions that you would never use, or you thought were unnecessary?
- Do you feel the security features are adequate in protecting your information? If not, what type of security measures would you like to see on the next version of the platform?
- How do you feel about the name of the platform?
- How do you feel about the logo of the platform?
- How did you feel about the language used in the platform? Did you find it to be too casual or too medically technical? If you were designing it, how might you change the language?
- Are there other contents that you would like to see on the next version of the platform? (HIV/AIDS education, statistics, services offered by certain physicians, option to connect with a peer navigator, etc.)?
- Were there parts of the platform that were difficult to understand? If so, which ones and what made them difficult? Is a list of LGBT-friendly physicians in your area sufficient to get you to seek necessary care? If not, what are the barriers that would keep you from going to see a doctor/get tested?

Concluding questions

- Would you use this function if it were available within Blued? Why or why not?
- Overall, do you think this type of platform could be effective in getting the MSM community to see physicians? Why or why not?
- What about helping you to find peer supporters?

Conclusion

- We greatly appreciate your participation; your feedback is essential to the success of this study.
- All viewpoints shared today are of great value to improving the mobile app, and ultimately helping more people get access to health care.
- We hope you have found this conversation to be engaging, and if there is anything you are unhappy with or wish to comment on, please feel free to reach out to us.
- At this point, I would like to reiterate that any comments published as a result of this study will be anonymous, and again we ask that you refrain from discussing fellow participant's comments outside of this room.
- Before you leave, please remember to leave your completed questionnaire and survey with me.
