# Supplementary material for: A Crowdsourced Physician Finder Prototype Platform for Men Who Have Sex with Men in China: Qualitative Study of Acceptability and Feasibility
Source: JMIR Public Health Surveill. 2019 Oct 8;5(4):e13027. doi: 10.2196/13027 (PMC6913768; doi:10.2196/13027)
Supplement: Multimedia Appendix 3 [file publichealth_v5i4e13027_app3.pdf]

Box 1 Acceptability

Quote 1
Participant 4 of group one in Shenzhen: if this (platform) comes into use, it will resolve (our concerns about) privacy. After all, many of us are afraid of going to public hospitals after getting these diseases because you are fearful of being judged by others around you. They may say that you are sexually indecent.
Quote 2
Participant 1 of group one in Shenzhen: If someone wants to ask about these (diseases), he may already have symptoms. This is when they are very anxious and afraid. This (platform) is a must.

Box 2 Specific functions of the platform - Physician consultation services

Quote 1
Participant 1 of group 2 in Guangzhou: If I suspect that I am infected, texts would be hard to fully explain the history. If they have audio consultation, I may just make a call.
Quote 2
Participant 4 of group 1 in Shenzhen: (I prefer) audio consultation. You can efficiently communicate. Physicians are too busy to read your messages one by one.
Quote 3
Participant 1 of group 1 in Guangzhou: I think one consultation section (on the platform) is enough and you can embed both text-image and audio-video in that.
Quote 4

Participant 3 group 1 in Shenzhen: These doctors are working in a hospital. When would they be able to respond to my questions? This may be a problem.

Participant 1: The physician who gave me his own contact information said it is difficult to provide a real-time reply.

Participant 3: He can only use his spare time to deal with our problems.

Quote 5

Participant 3 group 1 in Shenzhen: So it would be important to make appointments.

Participant 1: Yes, and it must be equal.

Participant 5: If I paid and you do not reply for long time, then this is meaningless.

Quote 6

Participant 1 of group 1 in Guangzhou: you need authorities to back up the platform. At least the physicians should be recognized by official authorities.

Quote 7

Participants of group 2 in Shenzhen: Which hospital is this doctor from? This is a quality control issue."

Quote 8

Participant 1 of group 1 in Guangzhou: In terms of physician consultations, they should focus on the type of illnesses that might affect our (MSM) self-esteem, or during the communication of certain illness, the doctor himself may be able to identify our (personal) information. Other services are unnecessary because it doesn't matter whether I disclose (sexual orientation) or not and nobody cares. But for anorectal, dermatological, or sexually transmitted diseases, they are likely to ask about your personal life.

Quote 9

Participant 1 of group 1 Shenzhen: Both STIs and HIV infection are like chronic illnesses. If you want cure or at least get the condition under control, you have to comply. It will be a long-term process, unlike common colds. Maybe this platform can provide continuous, follow-up services, and help facilitate patients complying to treatment.

Quote 10

Participant 2 of group 2 in Guangzhou: From the point of elderly men, this is necessary. I noticed that, on Blued, some people are live streaming for middle-age or elderly men to teach them how to maintain health. There is a market for middle-aged and elderly men because they not only have sexual needs but also have healthcare needs.

Box 3 Specific functions of the platform - Psychological support and consultations

Quote 1

Participant 1 of group 2 in Guangzhou: I think psychological problems are more important than physical symptoms. If he feels unhappy for a long time, he may develop depression.

Quote 2

Participant 1 of group 1 in Shenzhen: it is almost resourceless in this area. I have a boyfriend and he wants to move in with me, but I doubt myself and do not tell him the true reasons why I don't want to live with him. I have no one to talk to about this. Definitely not to the doctor who would be very busy and doesn't have that time to deal with these issues.

Quote 3

Participant 2 and 5 of group 1 in Shenzhen: we don't come out to doctors because they cannot help with mental issues, just physical problems. It doesn't make

sense to disclose (our sexual orientation).

Quote 4

Participant 3 of group 1 in Guangzhou: Professionals should be used for coming out to parents. When they say "you need to see a doctor" (meaning parents regard homosexuality as a disease), then you should bring them to gay-friendly physicians on this platform. So you can use this when your parents intend to bring you to a doctor, or when you develop real mental disorders.

Quote 5

Participant 1 of group 2 in Shenzhen: As you grow older, your family and relatives will urge you to get married and set up meetings with girls. You would then become very anxious and don't know what to do. I am more interested in how I can communicate with my family and how to educate them about this (homosexuality). To my mother, she may have absolutely no idea what homosexuality is. I wanted to talk to my Dad many times, but I don't know how to start the conversation.

Box 4 Box 3 Specific functions of the platform – sexual health education

Quote 1

participant 2, group 2 in Guangzhou: health education about sexual health can be added, like how to prevent HIV

Quote 2

participants 2 and 4, group 2 in Shenzhen: young people who just entered this circle are curious but less cautious about protecting themselves. They don't pay attention to the risk of unprotected sex. Sex education is too little, even in a developed city like Shenzhen.

Quote 3

Participant 2 of group 1 in Guangzhou: if this platform is only for dealing with diseases, I would only think about it when I was sick. But if you have educational information, such as HIV knowledge, I would use it more often because it is hard to find this somewhere else.

Quote 4

participant 2 of group 1 in Guangzhou: I had an HIV-positive boyfriend before, but I don’t know how I should live my daily life with him when I am negative. What I should do, what I should know and where I could find that information. This is almost resourceless.
